# Development of a Nanoemulgel for the Topical Application of Mupirocin

**DOI:** 10.3390/pharmaceutics15102387

**Published:** 2023-09-26

**Authors:** Bahjat Alhasso, Muhammad Usman Ghori, Simon P. Rout, Barbara R. Conway

**Affiliations:** 1 Department of Pharmacy, School of Applied Sciences, University of Huddersfield, Huddersfield HD1 3DH, UK; bahjat.alhasso@hud.ac.uk (B.A.); m.ghori@hud.ac.uk (M.U.G.); s.rout@hud.ac.uk (S.P.R.); 2 Department of Biological and Geographical Sciences, University of Huddersfield, Huddersfield HD1 3DH, UK; 3 Institute of Skin Integrity and Infection Prevention, University of Huddersfield, Huddersfield HD1 3DH, UK

**Keywords:** eucalyptus oil, eucalyptol, mupirocin, nanoemulgel, permeation, topical

## Abstract

Mupirocin (MUP) is an effective topical antibiotic with poor skin permeability; however, its skin permeability can be improved by a nanoemulsion formulation based on eucalyptus oil or eucalyptol. Despite this improvement, the nanoemulsion has limitations, such as low viscosity, low spreadability, and poor retention on the skin. To overcome these limitations, the aim of this study was to develop a nanoemulgel formulation that would enhance its rheological behaviour and physicochemical properties. The MUP nanoemulgel was prepared by incorporating a preprepared MUP nanoemulsion into Carbopol gel at a concentration of 0.75% in a 1:1 ratio. The nanoemulgel formulations were characterised and evaluated for their physicochemical and mechanical strength properties, rheological behaviour, and in vitro skin permeation and deposition, as well as antibacterial studies. Both nanoemulgels exhibited stability at temperatures of 4 and 25 °C for a period of 3 months. They had a smooth, homogenous, and consistent appearance and displayed non-Newtonian pseudoplastic behaviour, with differences in their viscosity and spreadability. However, both nanoemulgels exhibited lower skin permeability compared to the marketed control. The local accumulation efficiency of MUP from nanoemulgel after 8 h was significantly higher than that of the control, although there was no significant difference after 24 h. Micro-CT scan imaging allowed visualisation of these findings and interpretation of the deposited drug spots within the layers of treated skin. While there were no significant differences in the antibacterial activities between the nanoemulgels and the control, the nanoemulgels demonstrated superiority over the control due to their lower content of MUP. These findings support the potential use of the nanoemulgel for targeting skin lesions where high skin deposition and low permeability are required, such as in the case of topical antibacterial agents.

## 1. Introduction 

Nanotechnology is an attractive strategy for drug delivery and targeting, with considerable promising potential and desirable functional and advantageous features [1,2] to target specific sites of action with high therapeutic efficacy and reduce adverse reactions [3]. Nanoemulsions (NEs) have a large surface area, high entrapment efficiency for hydrophobic drugs, kinetic stability, solubilisation capability, high skin permeability, controlled release, and targetability as a drug carrier [4,5]. They have been developed for topical delivery of a range of actives, such as naproxen [6], curcumin [7], mupirocin [8], and tamoxifen [9]. However, low viscosity, low spreadability, and poor retention on the skin can limit their suitability [10]. A potential solution is to incorporate the nanoemulsion into a hydrogel base in order to thicken the formulation and improve its rheological behaviour and physicochemical properties [11].

Nanoemulgels are a novel topical formulation widely investigated in order to target various dermatological conditions, such as skin infections. They are suitable carriers for both hydrophilic and lipophilic drugs [12,13]. A nanoemulgel comprises a reservoir, which releases the drug quickly, increases its absorption, and enhances skin penetration [14]. By reducing surface tension and improving the rheological behaviour of nanoemulsions, nanoemulgels increase stability [15] and spreadability [16]. 

**Figure 1:**
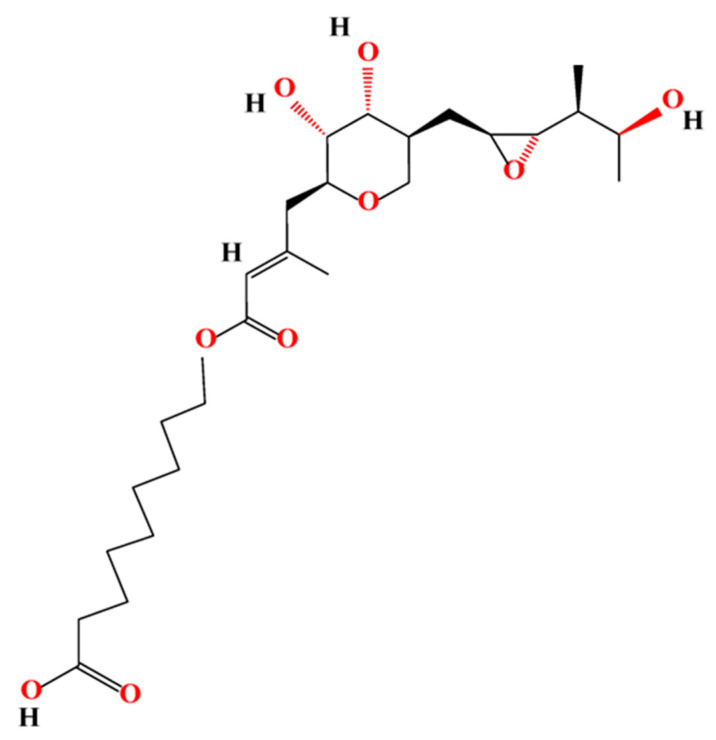
Chemical structure of mupirocin.

Mupirocin (MUP) is an effective broad-spectrum antibacterial agent used widely in the treatment of superficial topical infections [17] and inhibition of biofilm formation [18]. MUP is a natural analogue of isoleucyl adenylate, synthesised by soil bacteria, *Pseudomonas fluorescens* [19,20]. MUP, previously known as pseudomonic acid A, is composed of 9-hydroxynonanoic acid connected to monic acid by an ester linkage, as shown in Figure 1 [21,22]. 

MUP exhibits promising in vitro antibacterial activity against Gram-positive staphylococci, especially methicillin-resistant *S. aureus* (MRSA), and so it is used in the prophylaxis and treatment of infections caused by MRSA, such as skin, skin appendage, and mucosal membrane infections [23]. Moreover, most streptococci spp. and some Gram-negative bacteria, such as *Haemophilus influenzae* and *Neisseria* spp., are sensitive to MUP [24,25]. However, the therapeutic effect of MUP is hampered due to its short half-life (<30 min) and the emergence of bacterial resistance [26]. Furthermore, the therapeutic efficacy of MUP is impeded by metabolic inactivation and instability associated with its high plasma protein binding when administered parenterally [27,28]. Therefore, MUP use is limited clinically to the treatment of topical skin infections and the decolonisation of nasal carriage of *S. aureus*. Despite the topical antibacterial potential of MUP, its poor permeability into/through the skin has limited its clinical applications [27,28].

Terpene essential oils have been widely used in the development of topical formulations (dermal or transdermal dosage forms) due to their safety and efficacy [29]. Evidence shows that eucalyptus oil and its main component (eucalyptol) have promising antimicrobial and pharmaceutical activities [30,31,32]. In the same manner, the presence of eucalyptus oil or eucalyptol in a nanoemulgel of MUP might increase the therapeutic effect of the drug and enhance its permeability. To our knowledge, the current study is the first to report the use of nanoemulgels as a novel nanocarrier for MUP by incorporating an optimised MUP-loaded nanoemulsion into an appropriate gel, such as a Carbopol hydrogel. Several polymers were screened in order to choose the appropriate candidate, and nanoemulgels were formulated, characterised, optimised, and evaluated. In addition, in vitro permeation studies using Strat-M^®^ membrane and porcine skin, determination of drug deposition in skin, and antibacterial studies of MUP were carried out and compared with a marketed MUP cream.

## 2. Materials and Methods

### 2.1. Materials

MUP (purity > 98%) was purchased from Tokyo Chemical Industry UK Ltd. (Oxford, UK) and Discovery Fine Chemicals Ltd. (Leek, UK). Polyoxyethylene sorbitan monooleate (Tween^®^ 80), sorbitan mono oleic acid (Span^®^ 80), eucalyptol (EU) (purity 99%), eucalyptus oil (EO) (purity 100%), and absolute ethanol (purity ≥ 99.8) were all analytical grade and were purchased from Sigma Aldrich (Gillingham, UK). Acetonitrile (purity ≥ 99%), methanol (purity ≥ 99.5%), and ortho-phosphoric acid (85%) were all HPLC grade and were purchased from Sigma Aldrich (Gillingham, UK).

Carbopol 940 was purchased from Acros Organics B.V.B.A. (Geel, Belgium), HPMC K100 and xanthan gum from *Xanthomonas campestris* were purchased from Sigma Aldrich (Gillingham, UK), and Xantural^®^ 75 was purchased from CP Kelco (Leatherhead, UK). 

Ultrapure water was obtained from Barnstead Nanopure (Texas, TX, USA). Merck Strat-M^®^ membrane and B Braun^™^ hypodermic needles and adhesive tape (3M Transpore^®^) were purchased from Thermo Fisher Scientific (Warrington, UK).

### 2.2. Solubility Determination

MUP solubility was measured in different NE components by dissolving excess MUP in a defined volume of solvent at 25 ± 1 °C and shaking at 100 rpm for 72 h to achieve equilibrium. Samples were diluted and filtered through a 0.45 μm syringe filter before HPLC analysis to measure the dissolved MUP in each solvent [8]. 

### 2.3. HPLC Method

HPLC analysis of MUP was carried out using a Shimadzu HPLC (LC-10AT pump, LC-20AT autosampler, and UV-VIS (SPD-20AV) detector). An XTerra MS C18 Column (125 Å, 3.5 µm, 4.6 mm × 150 mm) was used for MUP separation, using an isocratic mobile phase consisting of phosphoric acid in water (pH 2.75 ± 0.05) and acetonitrile (60:40). The flow rate was 1 mL/min for 10 min in an injection volume (20 µL) maintained at 40 °C temperature and detected at 220 nm wavelength [8].

### 2.4. Preparation of Nanoemulsions

**Figure 2:**
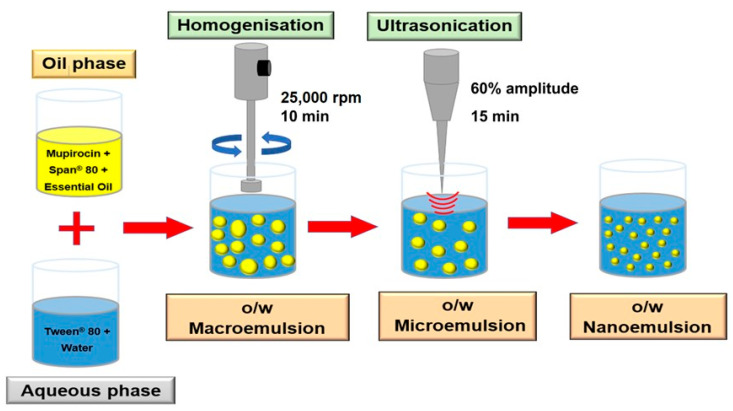
Schematic diagram for the nanoemulsion preparation.

The aqueous phase (Tween 80 in water) and the lipid phase (Span 80 in essential oil) were heated to 60 °C and combined. The emulsion was homogenised using a high-shear homogeniser (Ystral GmbH D-7801 Dottingen, X1020 homogeniser, Ballrechten-Dottingen, Germany) and an ultrasonic probe homogeniser (Model 3000MP Ultrasonic homogeniser, Biologics Inc., Manassas, VA, USA) for 15 min at 60% amplitude and cooling. The formulated nanoemulsion was characterised after cooling to 25 °C, and an optimised nanoemulsion was designed [8] and is shown in Figure 2.

### 2.5. Preparation of MUP-Loaded Nanoemulsions

The method was the same as outlined in Section 2.4, except MUP was dissolved in ethanol and mixed with essential oil. Span 80 was incorporated into the mixture after the removal of ethanol by rotary evaporation [8]. 

### 2.6. Measurement of Size, Polydispersity Index, and Zeta Potential of Nanoemulsions

A nanoemulsion sample was diluted in ultrapure water at a ratio of 1:3 and equilibrated for 60 s at 25 °C in order to use a Zetasizer (Malvern Nano ZS, Malvern, UK) to measure the droplet size and polydispersity index (PDI). Using a dip cell, 10 µL of nanoemulsion sample was diluted in 990 µL of ultrapure water to measure the zeta potential, following equilibration for 120 s at 25 °C. All measurements were in triplicate and expressed as the mean and standard deviation (SD).

### 2.7. Determination of Entrapment Efficiency (EE%)

The nanocarrier was dissolved in methanol to determine the amount of drug in the formulation, which was then filtered (0.45 µm) and analysed using HPLC. The amount of drug present was calculated as an encapsulation (incorporation) efficiency using Equation (1) [33,34]:

(1) $$\mathrm{Incorporation}\;\mathrm{efficiency}=\frac{\mathrm{Drug}\;\mathrm{quantity}\;\mathrm{in}\;\mathrm{nanocarrier}}{\mathrm{Initial}\;\mathrm{drug}\;\mathrm{quantity}}\times 100$$

### 2.8. Preparation of Hydrogel

As shown in Table 1, various hydrogel preparations were prepared using a dispersion method. Briefly, the defined polymer was dispersed slowly in deionised water under continuous stirring until homogenous gel formation. Triethanoleamine was added to neutralise the pH of gel, which had been left overnight to complete cross-linking, and gelation, as well as expel any trapped air bubbles within gel.

### 2.9. Selection of the Gel for Nanoemulgel Formulation

#### 2.9.1. Measurement of Viscosity of Hydrogel and Nanoemulgel

Hydrogel viscosity was measured using a Bohlin Gemini cone and plate rheometer (Malvern Instrument Ltd., Malvern, UK) at 25 °C, using shear ramp (0.1–100) s^−1^ in triplicate and expressed as mean (±SD). The same parameters were used for viscosity measurement of nanoemulgel and a control (Savlon Advanced Healing Gel^®^).

#### 2.9.2. Texture Analysis Profile

The mechanical properties of hydrogels, nanoemulgels, and the control gel were studied using texture analysis (TAXT2, Stable Micro Systems, Surrey, UK) equipped with a 5 kg load cell. The hydrogel was placed into the 20 mL vial and allowed to settle at 25 °C for 15 min. The formulation surface was rendered smooth, and the trigger force was set at 5 g for this test. The test was performed in 1 cycle, triplicated, and expressed as mean ± SD. The data were collected and analysed using Exponent Lite Express software, version 6.1.16.0, to determine various mechanical properties, such as firmness (hardness), consistency, cohesiveness, and work of cohesiveness (adhesiveness). Texture analysis can also indicate spreadability [35] by determining various parameters, such as force (g), distance (mm), and time (s) [36,37]. It was used to calculate the spreadability of the nanoemulgel formulations (with and without MUP).

(2) $$\mathrm{Spreadability}=\frac{\mathrm{Mass}\;\left(\mathrm{Force}\right)\;\left(g\right)\times \mathrm{Distance}\;\left(\mathrm{mm}\right)}{\mathrm{Time}\;\left(s\right)}$$

### 2.10. Preparation of Nanoemulgel with/without MUP

Nanoemulgels were prepared by mixing the nanoemulsion (with/without MUP) with hydrogel at a 1:1 ratio, under continuous slow stirring (125 rpm) at 25 °C until visually homogeneous product formation. 

### 2.11. Characterisation of Nanoemulgel

#### 2.11.1. Visual Examination

The organoleptic characteristics of formulated nanoemulgels, including colour, odour, phase separation, consistency, and homogeneity, were inspected visually. Phase separation was assessed by centrifugation at 5000 rpm and 10,000 rpm for 10 min [38]. 

#### 2.11.2. Determination of Particle Size, Polydispersity, and Zeta Potential

Nanoemulgel formulations were suspended in 5 mL of ultrapure water under the conditions outlined in Section 2.6 to determine the droplet size and polydispersity index (PDI). The zeta potential was measured by suspending 20 mg of nanoemulgel formulation in 3 mL of ultrapure water, using a dip cell under the conditions outlined in Section 2.6. All the measurements were triplicated and expressed as mean ± SD.

#### 2.11.3. Determination of the pH of Gel and Nanoemulgel

A small amount of formulation was placed on universal indicator paper and left for 30 s, then the colour was visually observed and compared with the colorimetric pH value scale to evaluate the result. The test was carried out in triplicate.

#### 2.11.4. Assessment of Spreadability of Nanoemulgel

A 0.5 g of gel or nanoemulgel was placed on a glass plate in a defined circle with a 2 cm diameter (D_1_). A second glass plate was placed over the first one. A 500 g weight was placed on the upper glass plate for 3 min. The diameter of the circle after the spreading (D_2_) of gel or nanoemulgel was measured in order to determine the degree of spreadability [39]. 

### 2.12. Thermodynamic Stability Study

Selected formulated nanoemulgels were subjected to both long-term and accelerated stability tests in order to determine the stability of nanoemulgel.

#### 2.12.1. Long-Term Stability Studies

The selected formulations were kept for 3 months at 4, 25, and 40 °C. The viscosity was measured in triplicate each month for three months, and the values were expressed as mean ± SD.

#### 2.12.2. Accelerated Stability Studies

The formulations underwent 6 heating–cooling cycles, with each cycle consisting of refrigeration at 4 °C and oven exposure at 40 °C for 48 h at each temperature. Samples were visually inspected for signs of instability. Stable samples from the heating–cooling cycle were then subjected to centrifugation at 3750 rpm for 5 h. Visual inspection was performed to detect any separation or cracking, which simulated the gravitational force experienced over one year [40].

### 2.13. In Vitro Permeation Studies of MUP through Strat-M^®^ Membrane and Porcine Skin

#### 2.13.1. In Vitro Permeation Studies of Nanoemulgel Using Strat-M^®^ Membrane

In vitro permeation studies through Strat-M membrane were carried out using Franz diffusion cells [8]. Briefly, the diffusion was carried out over an area of 2.5 cm^2^ and a 15 mL volume of the receiver chamber. The experiment was carried out at a temperature of 37 ± 1 °C, with magnetic stirring at 100 rpm for 24 h. The receiver chamber was filled with medium composed of methanolic phosphate buffered saline in a ratio of 1:1 at pH 7.4. The nanoemulgel was placed in the donor chamber, and an aliquot (0.5 mL) was withdrawn from the receiver chamber and substituted with the same volume of fresh medium at a regular interval of 1 h for 24 h. HPLC analysis of samples was carried out without dilution. 

#### 2.13.2. Preparation of the Skin and In Vitro Skin Permeation Studies of Nanoemulgel

Porcine ear skin (full thickness) was supplied by a local abattoir and was used fresh after excision or kept frozen at −18 °C for future use within one month.

In vitro skin permeation studies were carried out as outlined previously [8], using the same parameters mentioned in Section 2.13.1, except the diffusion area was 3.14 cm^2^. The experiment was stopped after 8 and 24 h, and the formulation residues were removed from the skin before tape stripping, with and without cyanoacrylate, to determine drug within the skin [41].

### 2.14. Qualitative Determination of MUP Deposited in Skin Using a Micro-CT Scan

Following diffusion studies (8 and 24 h), skin was removed from the Franz diffusion cell, cleaned thoroughly, dried for 1 h, and scanned using the micro-CT Nikon Metrology (Nikon XT H 225, Nikon Corp. Tokyo, Japan) with the following parameters: tungsten target, accelerating voltage of 75 kV, gun current, 107 µA without copper filter. The sample was rotated for 360° at a resolution of 1008 pixel by 1008 pixel per projection. The sagittal plane was used to view from the surface to the base of the skin sample, showing a clear view within the three planes. The recording process for the projections lasted for 2 h. The projected images were then reconstructed and analysed using CT-pro and VG Studio 3.0 Software. False colouring was used to distinguish between materials with different densities.

### 2.15. Antibacterial Testing 

A 24 h sub-culture of *S. aureus* (NCIMB 9518) and MRSA (NCTC 13142) was prepared on Mueller–Hinton Agar (MHA). The culture was added to tryptone soya broth to generate an emulsion of ~1 × 10^8^–1 × 10^9^ CFU/mL and 100 µL added to the surface of MHA plates. A sterile 5 mm corer was used to remove 3 wells from each agar plate and the wells filled with test product in triplicate, and each plate was duplicated (so, n = 6). Following incubation for 24 h at 37 °C, the zone of inhibition was measured. The negative controls were *S. aureus* and MRSA without drug.

### 2.16. Statistical Data Analysis

All the measurements and calculations were carried out in triplicate and were expressed as mean ± SD. Analysis of variance (ANOVA) was used to test all mean values using MS Excel 2019. The differences were considered as statistically significant if the *p* value was less than 0.05.

## 3. Results and Discussion

### 3.1. Preparation and Optimisation of Nanoemulgel

#### 3.1.1. Viscosity Measurement

The nanoemulsions were successfully formulated and characterised, as detailed in a previous publication [8]. Carbopol was selected as the best polymer, based on preliminary studies of rheological properties and compared with a commercial control, Savlon^®^ Advanced Healing Gel (see Supplementary Materials, Figures S1–S3 and Table S1). Generally, any increase in the concentration of polymer resulted in an increase in viscosity. This is exploited to retain formulations at the affected skin area; however, an excessive increase in the viscosity might have some disadvantages in the spreadability of the formulation on skin and hinder drug release from the formulations due to the complexity of cross-linking at higher concentrations [42]. Although Carbopol 1% hydrogel had a higher viscosity than other Carbopol (CBL) hydrogels, Carbopol 0.75% produced the most viscous nanoemulgel, which would have better potential for keeping the nanoemulsion within the nanoemulgel. This might enhance the stability of the nanoemulsion before application, offer better retention and penetration of skin, and impact the release kinetics. Therefore, Carbopol 0.75% was chosen for further investigation. 

The mupirocin nanoemulgel-based Carbopol (MUP-NEG CBL) 0.75% hydrogel was more viscous than the control at low shear, with the order being reversed at high shear, suggesting that the MUP-NEG CBL 0.75% hydrogel would be more resistant to flow and drip (sag) than the control gel. In addition, the layer it forms would be thinner than that formed by the control gel. The control could be applied more easily than MUP-NEG CBL 0.75%, but it might drip. 

The relationship between shear stress and shear rate is presented later in (Figure 8B). CBL-based nanoemulgel formulations tend to exhibit non-Newtonian shear thinning behaviour. The shear rate is used as a determinant for the measurement of the viscosity of the formulations, i.e., a change in the shear rate causes a change in the viscosity, depending on the type of the product. This is caused by breaking of the polyalkanyl esters or divinyl glycol linkage between Carbopol monomers (acrylic acid). This cross-linkage is responsible for the elevated viscosity of the nanoemulgel. A high shear rate in testing disentangles and aligns the polymer chains. Subsequently, the chains realign in the same direction of the strain, resulting in decreased viscosity [43]. This property is crucial for topical formulations and is helpful in avoiding dripping of the formulation on the finger or at the site of application and results in easy and uniform spreading of the formulation on the skin. 

#### 3.1.2. Texture Analysis 

**Figure 3:**
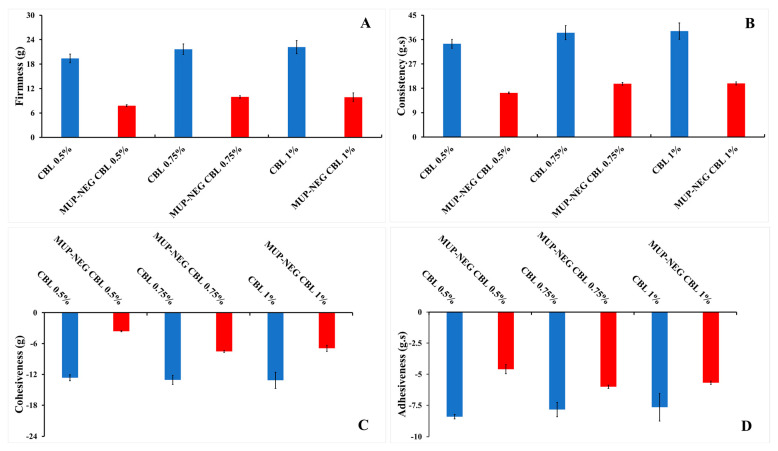
The effect of CBL concentration on the texture parameters of the gel and nanoemulgel formulations: (**A**) Firmness; (**B**) Consistency; (**C**) Cohesiveness; and (**D**) adhesiveness (mean ± SD, n = 3).

An increase in concentration of the polymer increases the firmness, consistency, and adhesiveness in a linear fashion for CBL (Figure 3); however, the cohesiveness of Carbopol gel was decreased in a linear fashion. All these parameters decreased when the Carbopol gel was combined with nanoemulsions. This might be due to the high content of the aqueous phase in the nanoemulsion, which affects the viscosity of the gel. These results agreed with the rheological studies and supported the use of Carbopol in the preparation of nanoemulgel formulations. Moreover, the combination of Carbopol with the nanoemulsion showed promising potential for the fabrication of the best nanoemulgel formulation, particularly with Carbopol 0.75% gel.

### 3.2. Physicochemical Characterisation of Nanoemulgel Formulations 

For further study, the nanoemulgel formulations were characterised using the following investigations.

#### 3.2.1. Organoleptic Properties

**Figure 4:**
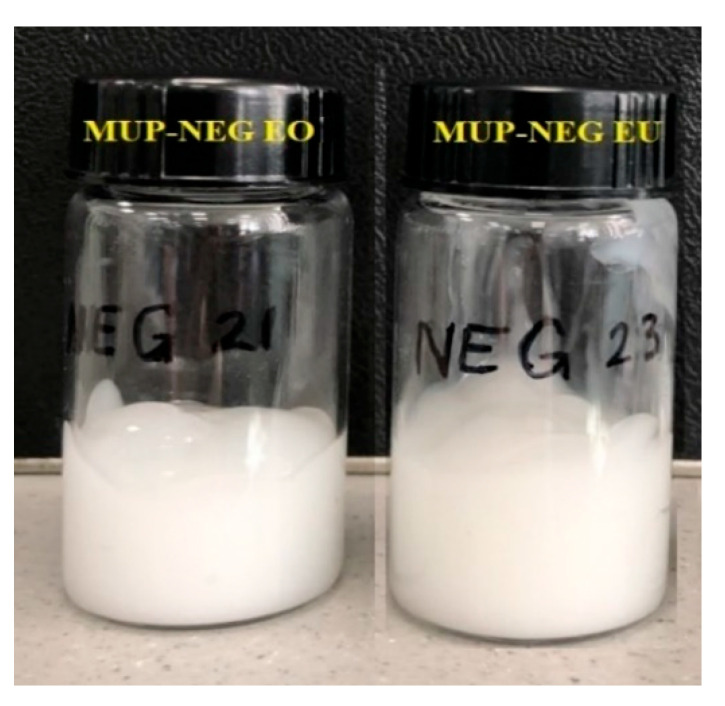
The physical appearance of nanoemulgel formulations: MUP-loaded nanoemulgel based on eucalyptus oil (MUP-NEG EO) and eucalyptol (MUP-NEG EU).

All the formulations were milky or off-white in colour. In addition, the formulations were homogenous, as shown in Figure 4.

#### 3.2.2. Measurement of Particle Size and Polydispersity Index (PDI) 

**Figure 5:**
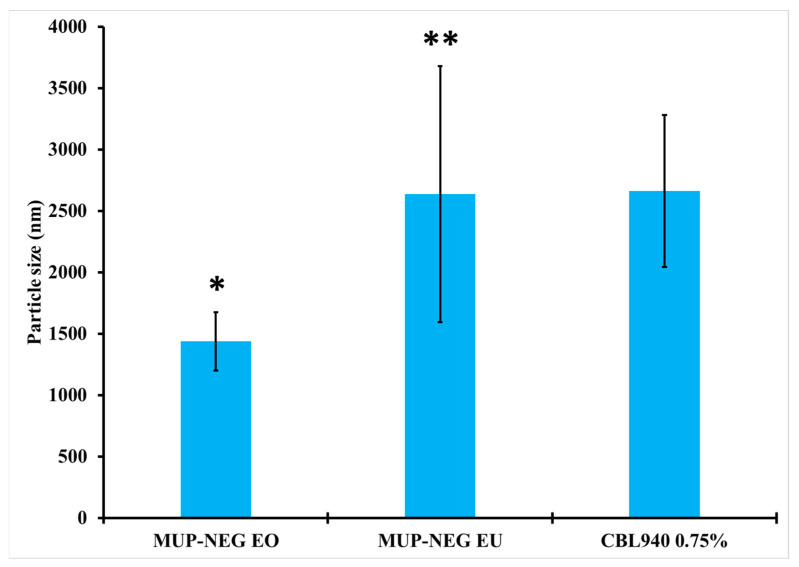
The particle size of nanoemulgel formulations and Carbopol 0.75% gel (mean ± SD, n = 3). * indicates significant difference compared to CBL940 0.75% gel; ** indicates no significant difference compared to CBL940 0.75% gel.

There was an increase in the particle size and PDI of the formulated nanoemulgel compared to the corresponding nanoemulsion. The increase in the particle size is due to measurement of the gelled Carbopol, which entrapped the nanodroplets within the polymeric matrix [44]. The particle size of MUP-NEG EU (MUP nanoemulgel based on EU) is significantly (*p* < 0.05) larger than that of MUP-NEG EO (MUP nanoemulgel based on EO) (Figure 5). 

**Figure 6:**
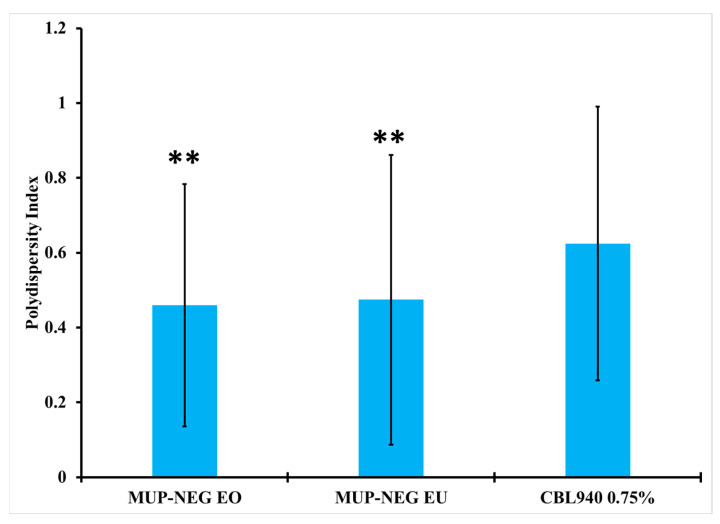
The PDI of nanoemulgel formulations and Carbopol 0.75% gel (mean ± SD, n = 3). ** indicates no significant difference compared to CBL940 0.75% gel.

The PDI of nanoemulgel formulations was less than Carbopol gel, although MUP-NEG EU was greater than MUP-NEG EO (*p* < 0.05). The addition of Carbopol gel into the nanoemulsion formulations did not result in a significant difference from the PDI of Carbopol gel without nanoemulsion (Figure 6).

#### 3.2.3. Determination of Zeta Potential 

**Figure 7:**
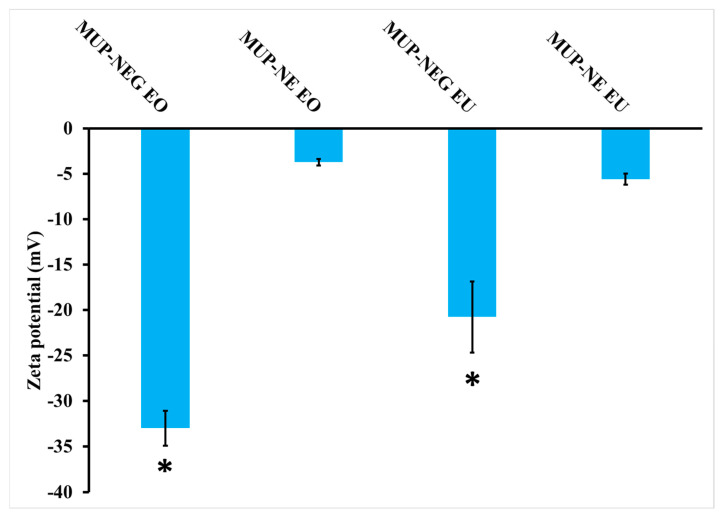
Zeta potential of nanoemulsion and nanoemulgel formulations (mean ± SD, n = 3). * indicates significant difference compared to CBL940 0.75% gel.

The magnitude of the zeta potential of nanoemulgels increased significantly (*p* < 0.05) compared to the corresponding nanoemulsion (Figure 7). The combination of nanoemulsion with Carbopol gel increased the magnitude of the zeta potential caused by adsorption of the polymer on the nanodroplets, providing steric stability, in addition to the location of the nanodroplets within the gel network, which limits their motion, increasing the stability of the product [14,45]. In addition, the high negative zeta potential originated from the carboxylic group on Carbopol. This functional group can interact electrostatically with the oil droplet-loaded MUP, offering higher colloidal stability [46]. Moreover, the thickening agent (Carbopol) can improve the stability of the nanoemulsion by increasing its viscosity [47]. 

#### 3.2.4. Measurement of pH 

All the nanoemulgel formulations had a pH of around 6 and were considered suitable to be used topically on the skin. 

#### 3.2.5. Determination of Viscosity

Carbopol 940 is a gelling agent used widely to thicken low-viscosity formulations, such as nanoemulsions. In addition, it interacts with certain surfactants to change the rheological properties, and the physical status of the nanoemulsion formulations changed from low-viscosity liquids to thick gels. This addressed several limitations of nanoemulsions, such as low viscosity, low spreadability, and poor dermatological retainability.

**Figure 8:**
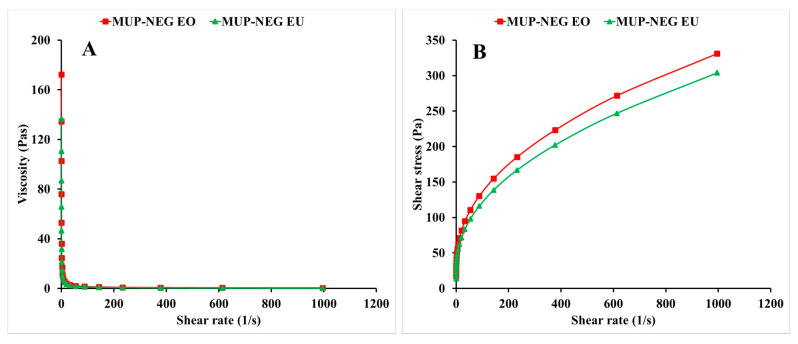
The viscosity–shear rate curve (**A**) and the shear rate–shear stress curve (**B**) of the nanoemulgel formulations (mean ± SD, n = 3).

Viscosity was determined at a shear rate of 100 s^−1^, and the viscosities for MUP-NEG EO and MUP-NEG EU were 134.53 ± 3.69 and 110.53 ± 3.69 Pas, respectively (Figure 8A), statistically different from each other. In addition, the nanoemulgels exhibited non-Newtonian behaviour (pseudoplastic shear thinning) (Figure 8B). An increase in the shear rate caused thinning of the nanoemulgel formulation, which is important for the spreading of topical formulations.

#### 3.2.6. Determination of Spreadability

The diameter of the formulation spread was 37 ± 1 and 38.3 ± 1.53 mm for MUP-NEG EO and MUP-NEG EU, respectively. The results showed good spreadability and agreed with the results reported by Almostafa, Elsewedy [48]. No significant differences were found in the spreadability among the nanoemulgel formulations (*p* < 0.05). 

**Figure 9:**
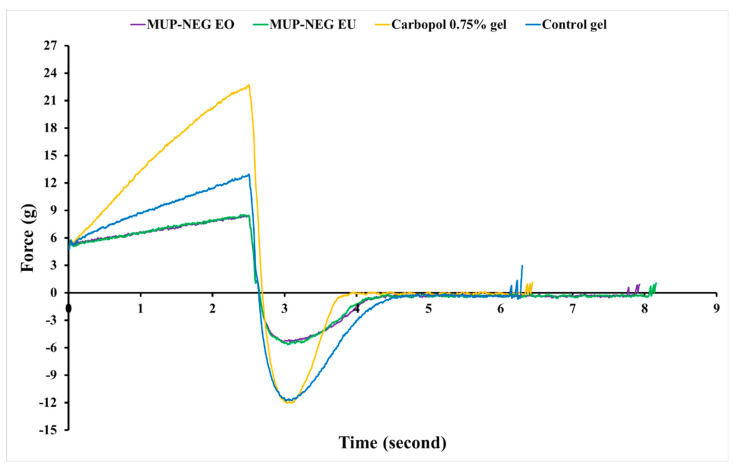
The texture profile analysis (force vs. time) for nanoemulgel formulations, Carbopol 0.75% gel, and control gel (n = 3).

The mechanical properties of the formulated nanoemulgels were analysed using a texture analyser, and the data are depicted in Figure 9. The firmness (hardness), cohesiveness, consistency, and adhesiveness of the optimised nanoemulgel formulations, Carbopol 0.75% gel, and the control gel were measured and are tabulated in Table 2. 

The firmness and consistency were similar for nanoemulgel formulations compared to the control gel and were firm enough to be applied to the skin. As this parameter is indirectly proportional to the spreadability, formulations with higher firmness require stronger application forces to make the formulation flow easily on the skin. The consistency of nanoemulgel formulations, a parameter directly related to the viscosity, was significantly lower than the control. Despite this, the nanoemulgel formulations were shown to be a viscous product with non-Newtonian behaviour, as represented in Figure 8B [49,50].

Carbopol gel had a similar cohesiveness to the control; however, the cohesiveness of the nanoemulgel formulations was significantly lower than the control and Carbopol gel (*p* < 0.05). Despite this, the nanoemulgel formulations were still able to be distorted easily due to weak internal bonds within the nanoemulgel structure, which would result in a negative cohesive force. This is desirable for formulations designed for topical application. The adhesiveness of nanoemulgel formulations was lower than Carbopol gel and the control (*p* < 0.05). As the nanoemulsion comprises 84.6% water, this decreases the gel viscosity, increasing the flow rate of nanoemulgel and, in turn, reducing the adhesiveness and sticking ability to the surface of the material (container). This parameter reflects the surface properties of nanoemulgels and depends on the viscoelasticity of the gel, as well as the adhesive and cohesive forces. Overall, the texture of the formulated nanoemulgels was soft and with more adhesiveness than the control [51,52]. 

Equation (2) [36,37] was used to calculate of spreadability of the nanoemulgel formulations (with and without MUP) (Table 3). 

In general, the spreadability of a formulation is indirectly proportional to its viscosity [38]. NEG EU was the most spreadable (28.17 ± 0.88 g·mm/s) compared to the other formulations (blank nanoemulgels, MUP-NEG EO, and the control gel). However, the difference in the viscosity of MUP-loaded nanoemulgel formulations did not have a significant effect on the spreadability of these formulations. Inclusion of drug within the nanoemulsion and interactions of the nanoemulsion with the hydrogel may dominate. This can be attributed to the effect of the formulation composition on the shearing force and magnitude [53,54,55]. MUP-loaded nanoemulgel formulations had relatively similar spreadability values; however, the control was more spreadable than the MUP-loaded nanoemulgels (*p* < 0.05). Despite this, the spreadability of the MUP-loaded nanoemulgel formulation was enough for the effective topical application on the skin, as reported by various studies [48,56]. 

### 3.3. Thermodynamic Stability Studies 

#### Long-Term Stability Studies 

Stability studies investigated the effect of storage temperature and the duration of storage on the viscosity of the nanoemulgel formulations. In addition, the organoleptic properties (colour and odour), pH, and any signs of separation were inspected, measured, and monitored.

**Figure 10:**
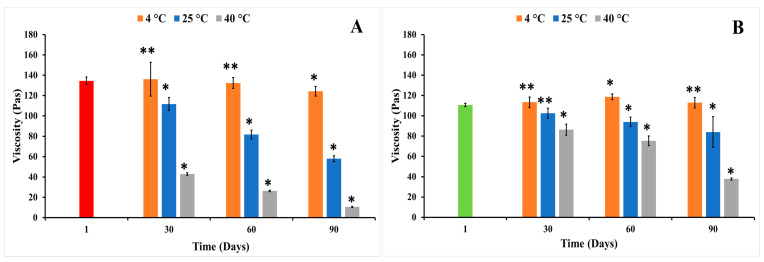
The viscosity of individual nanoemulgel formulations at 4, 25, and 40 °C in long-term stability study for 3 months: (**A**) MUP-NEG EO, (**B**) MUP-NEG EU (mean ± SD, n = 3). * indicates significant difference compared to the same nanoemulgel at first day of formulation; ** indicates no significant difference compared to the same nanoemulgel at first day of formulation; red and light-green bars represent MUP-NEG EO and MUP-NEG EU, respectively, at the first day of formulation at 25 °C.

In general, elevation of the storage temperature resulted in a reduction in the viscosity of nanoemulgel formulations; however, this effect might be influenced by the composition of the formulations. Storage at 40 °C (heating) significantly (*p* < 0.05) reduced the viscosity of both MUP-NEG EO and MUP-NEG EU compared to the same formulation at 25 °C (Figure 10A,B). This disparity increased with storage time. This can be attributed to the similarity between EO and EU, with EU comprising > 85% of the EO composition. There were no significant changes in the organoleptic properties of formulations, except MUP-NEG EO. The viscosity of MUP-NEG EO decreased significantly (*p* < 0.05) after 1 month at 40 °C, liquifying and slightly separating. A similar result was reported by Contreras, Diéguez [57] and may be due to the heating process inducing polymer–solvent and/or polymer–polymer interactions, disrupting the gel structure [58]. 

The duration of storage was also shown to affect the viscosity of the nanoemulgel formulations, illustrated for formulations stored at 25 °C. The viscosity of MUP-NEG EO and MUP-NEG EU decreased significantly (*p* < 0.05) after 3 and 2 months, although MUP-NEG EU looked stable, with a non-significant (*p* > 0.05) reduction in viscosity after 1 month of storage at 25 °C, as shown in Figure 10A,B.

At 4 °C (cooling) temperature, the viscosity of MUP-NEG EU was increased non-significantly (*p* > 0.05) over 2 months, as presented in Figure 10B, whereas the viscosity of MUP-NEG EO had irregular behaviour, depending on the duration of storage (Figure 10A). 

Thus, the stability of the nanoemulgel formulations represented by the acceptable viscosity was affected by the storage temperature and the duration of storage and the composition of the formulation including oil, surfactant, and polymer. 

**Figure 11:**
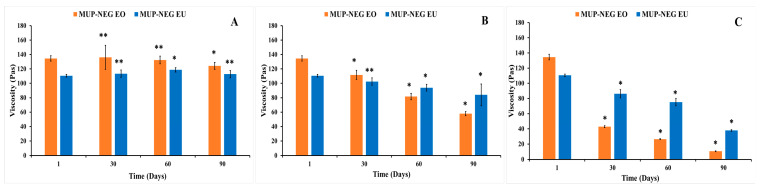
The viscosity of the nanoemulgel formulations in a long-term stability study for 3 months at (**A**) 4 °C, (**B**) 25 °C, and (**C**) 40 °C (mean ± SD, n = 3). * indicates significant difference compared to the same nanoemulgel on the first day of formulation; ** indicates no significant difference compared to the same nanoemulgel on the first day of formulation. Accelerated stability studies.

The study also indicated that MUP-NEG EO was the least stable formulation at 25 °C after 3 months. The reduction of the viscosity of MUP-NEG EO and MUP-NEG EU is directly proportional with the storage duration at 25 °C over 3 months (Figure 11B). The reduction in viscosity at 40 °C (Figure 11C) is because of the impact of elevated temperature and loss of dissolved gases altering pH and destabilising the formulation. 

The increase in viscosity of nanoemulgel formulations at 4 °C is less predictable and regular (Figure 11A). The viscosity of MUP-NEG EO and MUP-NEG EU both increased significantly (*p* < 0.05) after 3 and 2 months of storage, respectively, at 4 °C. 

Table 4 summarises the success of both nanoemulgel formulations for the centrifugation and heating–cooling cycles. 

The changes in the storage temperature between 4 and 40 °C during the accelerated stability test did not result in any signs of instability of the formulations, indicating the suitability of Carbopol 940 gel for further study. 

### 3.4. In Vitro Permeation Studies of MUP through Strat-M^®^ Membrane and Porcine Skin

#### 3.4.1. In Vitro Permeation Study of MUP from Nanoemulgel Formulations Using Strat-M^®^ Membrane

The following permeation parameters were estimated in this study: lag time (tlag), steady state flux (Jss), permeability coefficient (Kp), cumulative drug permeation over 24 h (Jmax or Q24), and enhancement ratio (ER), as shown in Table 5.

**Figure 12:**
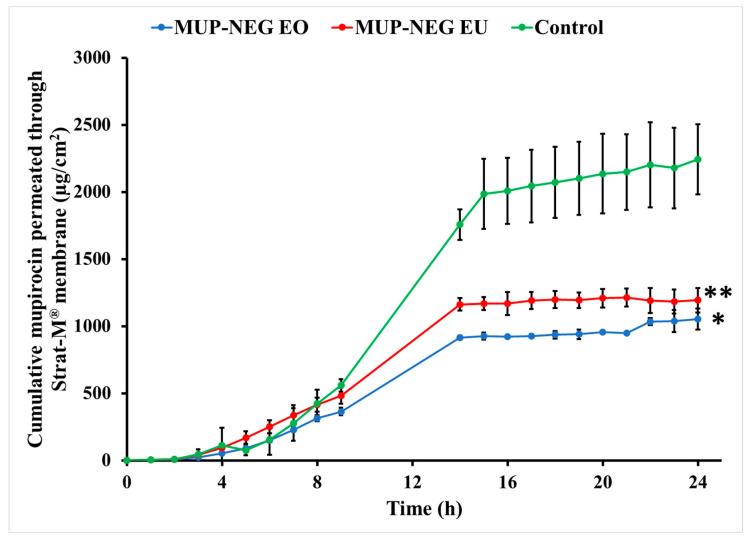
Cumulative in vitro permeation study of nanoemulgel formulations and control using Strat-M^®^ (mean ± SD, n = 3). * indicates significant difference compared to the control; ** indicates no significant difference compared to the control.

**Figure 13:**
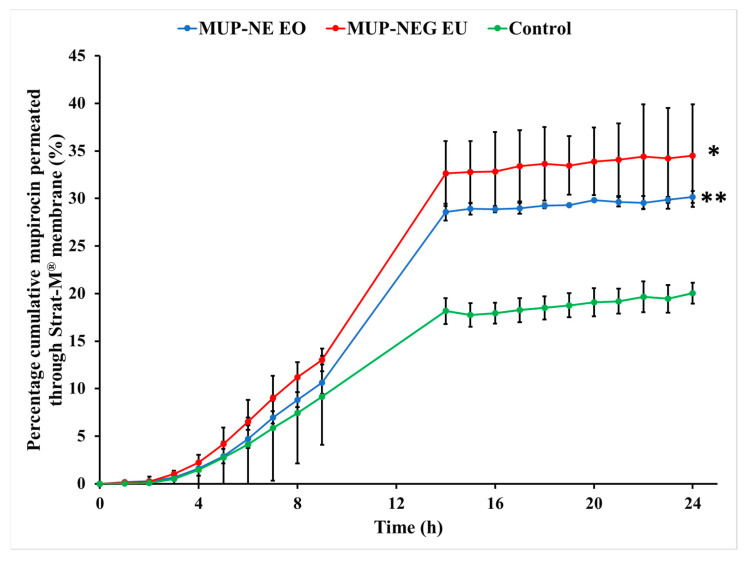
Cumulative percentage study of nanoemulgel formulations and the control using Strat-M^®^ (mean ± SD, n = 3). * indicates significant difference compared to the control; ** indicates no significant difference compared to the control.

The nanoemulgel formulations enabled quicker permeation with higher permeability coefficients than the control cream, a marketed Bactroban^®^ cream (2% *w*/*w* mupirocin). Conversely, the nanoemulgel formulations had lower flux and permeation enhancement ratios than the control. Although the nanoemulgel formulations permeated in higher amounts than the control, the amount of MUP permeated from the control is greater than that from the nanoemulgel formulations (Figure 12). Since the artificial membrane (Strat-M^®^) reflects the release process more than the actual permeation of drug [59], drug release from the nanoemulgel formulations is greater than the control (Figure 13). This could be why the nanoemulgel formulations reach a plateau stage faster than the control. Moreover, the higher concentration of MUP in the control might enhance permeation of the drug by acting as a reservoir, which prolongs the permeation period by maintaining a high concentration gradient of drug [60]. 

Although the concentration of MUP is higher in the control, there was more permeation of MUP from the nanoemulgel formulations after 24 h, likely due to the increase in thermodynamic activity and enhanced partitioning of the solubilised drug within the formulation [61]. In addition, the viscosity of nanoemulgel is less than the control cream, and inclusion of essential oils might also enhance the diffusivity of the drug through the membrane [62]. 

#### 3.4.2. In Vitro Skin Permeation Study of MUP from Nanoemulgel Formulation

Permeation parameters were determined as shown in Table 6.

**Figure 14:**
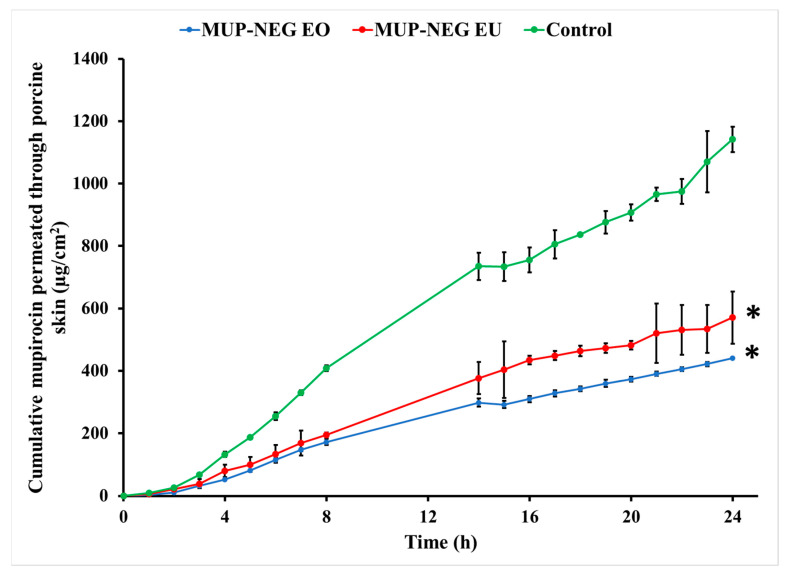
In vitro skin permeation of nanoemulgel formulations and the control (mean ± SD, n = 3). * indicates significant difference compared to the control.

Permeation of MUP from the control cream is significantly greater than the nanoemulgel formulations (*p* < 0.05), as represented in Figure 14. The gel matrix of Carbopol 940 with its small-size mesh delays the release of MUP, which would reduce absorption into the systemic circulation in vivo. In addition, drug diffusion from the formulation is reduced, maintaining an effective concentration of MUP locally. Similar results were reported by Harwansh, Patra [63] during comparison of a nanoemulgel formulation of glycyrrhizin with a conventional gel and nanoemulsion of the same drug. Also, Wavikar and VaviaWavikar and Vavia [64] reported a similar effect for a nanolipidgel of terbinafine. 

Generally, the lower amount permeated through the skin can be considered an indicator for the higher deposition of drug from in the skin, suggesting nanoemulsions can target the skin. The higher release from nanoemulsions compared to the cream control also supports this. The adhesive tape stripping method was used in order to confirm these results by examining drug deposition in the skin.

### 3.5. Quantification of MUP in Skin

#### 3.5.1. Quantitative Method: Differential Stripping Techniques

**Figure 15:**
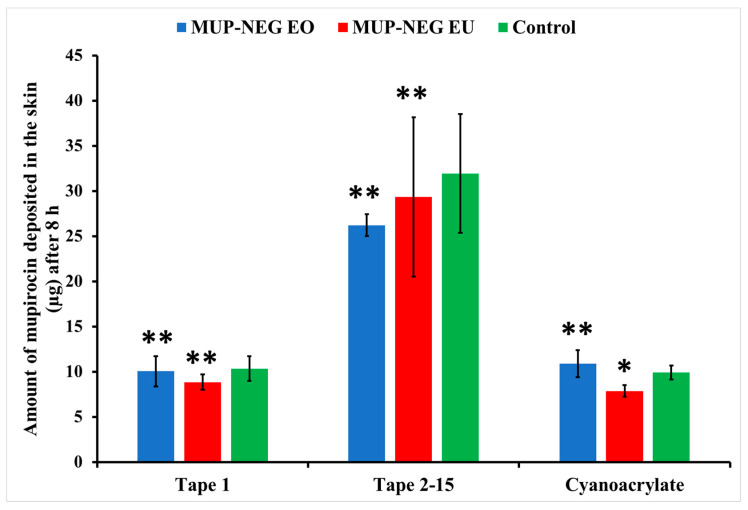
The amount of MUP in the upper parts of skin from nanoemulgel formulations and the control after 8 h (mean ± SD, n = 3). * indicates significant difference compared to the control; ** indicates no significant difference compared to the control.

**Figure 16:**
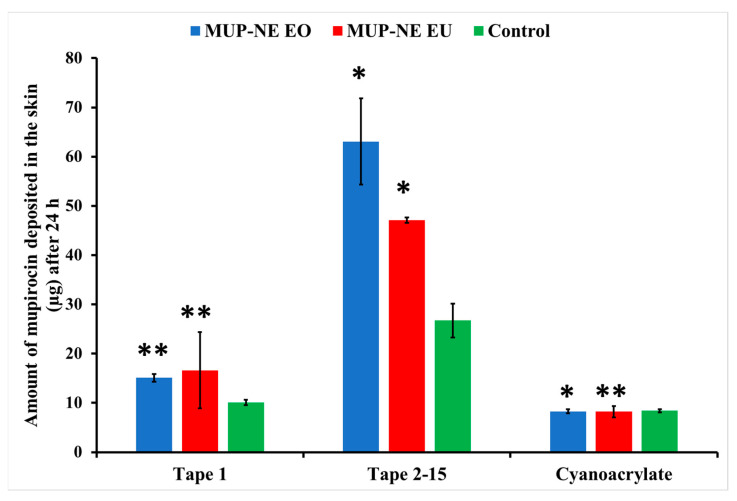
The amount of MUP penetrating the upper part of skin from nanoemulgel formulations and the control after 24 h (mean ± SD, n = 3). * indicates significant difference compared to the control; ** indicates no significant difference compared to the control.

Figure 15 and Figure 16 show deposition of the drug as the residual of the formulation on the skin and in the stratum corneum, represented by Tape (1) and (2–15), respectively, in addition to the cyanoacrylate biopsy, which represented the deposited drug in hair follicles after 8 and 24 h. After 8 h, there was no significant difference in the amount of MUP in the upper part of the skin (Tapes 2–15) compared to the control, except for MUP-NEG EU, which showed a significant reduction in deposition of MUP in the hair follicles as compared to the control, as represented by cyanoacrylate biopsy (Figure 15). A potential reason could be the high initial permeation rate due to the high concentration of drug at the donor compartment, which is a driving force for permeation. A similar effect was reported by Harwansh, Patra [63].

After 24 h, MUP-NEG EO and MUP-NEG EU showed a significant increase in the amount of MUP compared to the control (*p* < 0.05), represented by tapes 2–15 (Figure 16). In addition, the similarity in the composition between MUP-NEG EO and MUP-NEG EU due to the high percentage of EU in EO might also increase the effect of gel on the deposition of MUP within the skin layers.

**Figure 17:**
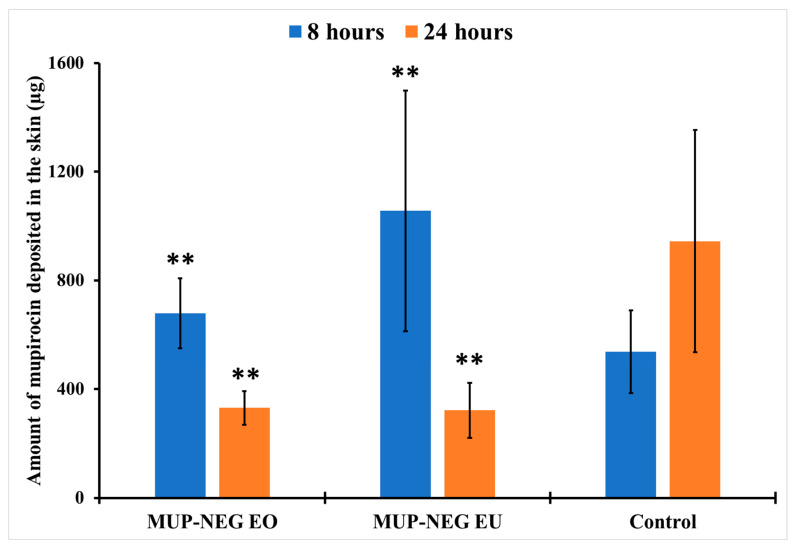
The amount of MUP deposited in the deeper part of skin from nanoemulgel formulations and the control (mean ± SD, n = 3) after 8 and 24 h. ** indicates no significant difference compared to the control.

The amount of MUP deposited in the skin after 8 h is not significantly different between the nanoemulgels and the control (*p* > 0.05) (Figure 17), although less MUP was deposited within the skin compared to the control after 24 h. This reduction in the drug deposition was considered statistically non-significant (*p* > 0.05).

Local accumulation efficiency (LAC) was used to explore these findings further to compare the accumulation of the antibacterial agent within the skin. LAC was calculated using Equation (3):

(3) $$\mathrm{Local}\;\mathrm{accumulation}\;\mathrm{efficiency}\;\left(\mathrm{LAC}\right)=\frac{\mathrm{Amount}\;\mathrm{of}\;\mathrm{drug}\;\mathrm{accumulated}\;\mathrm{within}\;\mathrm{the}\;\mathrm{skin}}{\mathrm{Amount}\;\mathrm{of}\;\mathrm{drug}\;\mathrm{permeated}\;\mathrm{through}\;\mathrm{the}\;\mathrm{skin}}$$

After 8 h, both nanoemulgel formulations had a significantly higher LAC (*p* < 0.05) compared to the control, as shown in Table 7. This result agreed with the reports of Harwansh, Patra [63] and Zheng, Ouyang [65]. The increase in the skin deposition of MUP might be mainly due to the presence of Carbopol 940 gel in nanoemulgel formulations. The reduction in the concentration of MUP in the external phase of nanoemulgel resulted in MUP diffusing from the internal reservoir to replace it. This process resulted in increasing in the Carbopol portion in the gel matrix, leading to an increase in drug deposition in the skin and a decrease in the transdermal permeation. In addition, the effect of EU in the depositing of MUP in the skin layers offers an advantageous feature for the nanoemulgel in the depositing of MUP in the skin, represented by the LAC values of each formulation. The high content of EU, with its ability to modify the skin properties, may result in the effective interaction of EU with the skin and deposition of the drug within the layers of skin. Moreover, the synergistic effect between nanoemulsion and Carbopol 940 hydrogel (permeation enhancement by nanoemulsion and retention time prolongation by Carbopol gel) might also be another reason to be considered. 

After 24 h, the LAC for nanoemulgels was similar to the control (*p* > 0.05) (Table 7). The increase in the concentration gradient of the drug in the skin due to deposition after 8 h resulted in increasing a driving force for transdermal permeation of the drug [66]. This might increase the flux rate and permeation through the skin at the expense of deposition in the skin. This effect occurred simultaneously with depletion of the driving concentration of MUP in the donor chamber, resulting in the reduction in the skin deposition of MUP. A similar result was reported by [63,64].

#### 3.5.2. Qualitative Method: Micro-CT 

**Figure 18:**
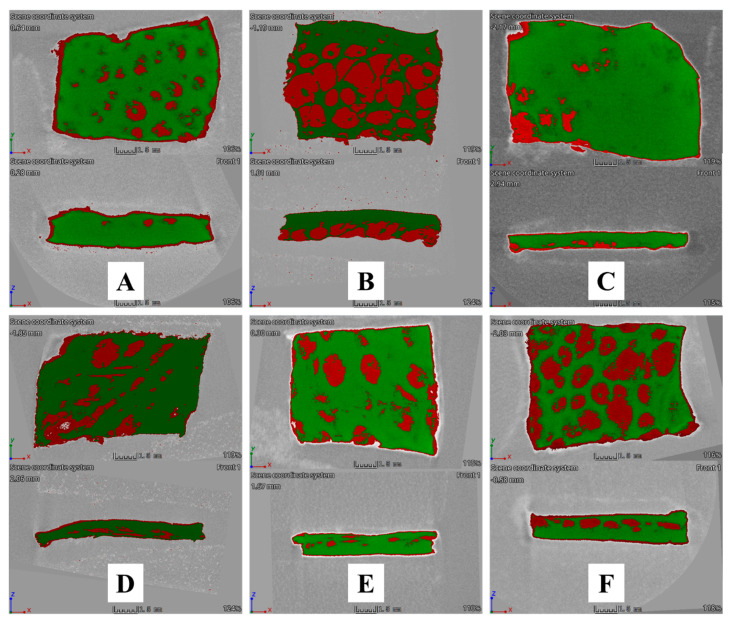
Cross-sectional Micro-CT scan images of porcine skin after (1) 8 h of application: (**A**) MUP-NEG EO, (**B**) MUP-NEG EU, and (**C**) Control cream and (2) 24 h of application: (**D**) MUP-NEG EO, (**E**) MUP-NEG EU, and (**F**) Control cream. Note: Red and green colour represent MUP and skin, respectively.

Micro-CT was used to visualise MUP penetrated and retained within the skin and support the quantitative results. Figure 18A–C show the visual presentation of MUP-penetrated skin after 8 h of skin permeation study. It shows larger red spots (MUP) distributed throughout the skin samples (green) as compared to red spots represented by Figure 18B,C. This indicated that the penetrated amount of MUP from different formulations occurred in an order of MUP-NEG EU > MUP-NEG EO > Control. This finding agreed with the quantitative results obtained from the adhesive tape stripping method. 

Figure 18D–F show the visual presentation of MUP-penetrated skin after 24 h of skin permeation study. Figure 18F shows an image crowded with red spots, confirming more MUP within the skin, while Figure 18D,E have less red colouring than the control. All the qualitative results depicted by micro-CT scan agreed with the quantitative results obtained from the adhesive tape stripping method, which showed the formulation in the following order: Control > MUP-NEG EO > MUP-NEG EU.

The micro-CT can image in 3D on a small scale with very high resolution and visualise the internal structure of the skin without sample destruction. However, it has a limitation in needing a contrast agent to overcome the low contrast of soft tissue and the difficulty differentiating the different skin layers. 

### 3.6. Evaluation of the Antibacterial Activity of MUP Nanoemulgels 

The zone of inhibition for nanoemulgels with/without MUP and the marketed product (Bactroban^®^ cream) are summarised in Table 8.

Based on the inhibition zone, there was no significant difference (*p* > 0.05) between the nanoemulgels and the control in their antibacterial activity against *S. aureus* and MRSA. However, the loading of MUP in the nanoemulgel is about half that of the marketed control; thus, the nanoemulgels improved the antibacterial activity of MUP against both strains of staphylococci. Various factors contribute to explain the reasons of this effect. 

Firstly, the small size of the nanoemulsion provides the product with a large surface area, which may help in the enhancement of penetration of the drug and, in turn, its activity [67]. This agrees with findings by Marslin, Selvakesavan [68], who succeeded in improving the antibacterial activity of *Withania somnifera* cream incorporated with silver nanoparticles.

The second cause of enhancement in the antibacterial activity of MUP might be related to the use of essential oils (EO or EU) within the nanoemulgel. These substances are well recognised for their anti-microbial activity against Gram-positive bacteria, such as *S. aureus* and MRSA, and may act synergistically with the MUP. Many studies have attributed the antibacterial effect of EO to its terpene components, particularly EU [69,70,71,72]. In addition to EU, EO contains components such as α-pinene, cuminylaldehyde, limonene, α-phellandrene, p-cymene, trans-pinocarveol, terpinen-4-ol. α-phellandrene, and terpinene-4-ol, which have shown promising antibacterial effects against *S. aureus* [73] by disruption of the cellular integrity, inhibition of ion transportation, and respiration [74].

On the other hand, prolonged contact of the nanoemulgel with bacteria resulted in an increase in the concentration of drug penetrating into bacteria [68]. Assali, Zaid [75] attributed an improvement in the antibacterial activity of ciprofloxacin to the effect of the single-walled nanotube. This formulation led to aggregation of antibacterial agents around the microorganism, extending the duration and penetration of the antibacterial agent into bacterial cells. Mayaud, Carricajo [76] reported that contact times for various essential oils with various bacteria of more than 5 min were required to inhibit bacterial growth.

## 4. Conclusions

In summary, a nanoemulgel formulation was successfully developed by adding Carbopol 940 (0.75%) hydrogel to the optimised nanoemulsions based on two essential oils (EO and EU), separately at a ratio of 1:1. Stable, smooth, homogenous, and consistent nanoemulgel formulations were obtained at 25 °C. Both nanoemulgels were stable, with a higher zeta potential value as compared to the corresponding nanoemulsion. Both nanoemulgels exhibited non-Newtonian pseudoplastic shear thinning behaviour with non-significant differences in the viscosity and approximately the same spreadability value (~17 g·mm·s^−1^). Both nanoemulgels remained stable at 4 and 25 °C for 3 months; however, at 40 °C, MUP-NEG EO tended to liquify and lost its consistency. The skin permeability of MUP from the nanoemulgel formulations was lower than the marketed cream control, while the LAC results indicated higher skin deposition of MUP from the nanoemulgel formulations after 8 h compared to the control, with no significant difference after 24 h. The micro-CT scan confirmed this finding, enabling a visual interpretation of the deposited drug within the layers of treated skin. Although the antibacterial study findings have shown no significant difference between the MUP nanoemulgels and the control, the lower amount of MUP loaded into the nanoemulgel formulations indicates their superiority over the control.

These findings indicated the promising potential of the nanoemulgel formulations for the topical delivery of antibacterial drugs, particularly when targeting skin lesions requiring high skin deposition and low permeability. This requires further investigation in order to determine whether the drug is primarily deposited in the dermis or in the epidermis. 

## Figures and Tables

**Table 1:** Details of hydrogel formulation of different polymers.

Polymer	Concentration (% *w*/*w*)	Mixing Rate (rpm)	Processing Temperature (°C)	Triethanolamine (mL)
Carbopol 940	0.5 0.75 1	400	25	0.15
Xanthan gum	1 1.5 2 2.5	400	40	0.25
HPMC	1 2	400	40	0.25

**Table 2:** The mechanical properties of the nanoemulgel formulations, Carbopol 0.75% gel, and the control (mean ± SD, n = 3).

Formulations	Mechanical Properties			
	Firmness (g)	Cohesiveness (g)	Consistency (g·s)	Adhesiveness (g·s)
MUP-NEG EO	8.55 ± 0.32	−5.53 ± 0.04	17.80 ± 0.52	−5.72 ± 0.05
MUP-NEG EU	8.67 ± 0.21	−5.72 ± 0.26	17.98 ± 0.29	−5.95 ± 0.70
Carbopol 0.75% gel	21.66 ± 1.30	−13.07 ± 0.87	38.54 ± 2.62	−7.84 ± 0.57
Control gel	12.99 ± 0.39	−11.91 ± 0.39	24.22 ± 0.67	−12.03 ± 0.32

**Table 3:** Spreadability of nanoemulgel (with and without MUP) and the control (mean ± SD, n = 3).

Formulation	Spreadability (g·mm/s)	Viscosity (Pas)
NEG EO	20.14 ± 0.99	252.97 ± 11.67
NEG EU	28.17 ± 0.88	68.47 ± 2.19
MUP-NEG EO	17.12 ± 0.64	134.53 ± 3.69
MUP-NEG EU	17.26 ± 0.46	110.53 ± 1.69
Control	25.97 ± 0.82	79.13 ± 1.61

**Table 4:** Summary results of accelerated stability studies of nanoemulgel formulations.

Formulation	Centrifugation	Heating and Cooling	
		4 °C	40 °C
MUP-NEG EO1	Stable (Pass)	Stable (Pass)	Stable (Pass)
MUP-NEG EU	Stable (Pass)	Stable (Pass)	Stable (Pass)

**Table 5:** The permeation parameters for nanoemulgel formulations and control using Strat-M^®^ membrane (mean ± SD, n = 3).

Parameters	MUP-NEG EO	MUP-NEG EU	Control
t_lag_ (h)	2.06	1.89	2.64
J_max_ (µg/cm^2^)	1053.41 ± 78.82	1194.07 ± 91.96	2242.79 ± 262.17
J_ss_ (µg/cm^2^/h)	53.53 ± 2.89	64.92 ± 7.52	112.42 ± 3.75
K_p_ (×10^−4^ cm/h)	55.69 ± 0.69	60.67 ± 0.22	107.14 ± 2.73
Enhancement ratio (ER)	0.47	0.53	1

**Table 6:** The permeation of nanoemulgel formulations and the control using porcine skin (mean ± SD, n = 3).

Parameters	MUP-NEG EO	MUP-NEG EU	Control
t_lag_ (h)	2.08	1.47	1.86
J_max_ (µg/cm^2^)	440.43 ± 24.33	570.97 ± 83.67	1141.61 ± 40.45
J_ss_ (µg/cm^2^/h)	16.39 ± 1.09	26.46 ± 2.44	39.32 ± 4.27
K_p_ (×10^−4^ cm/h)	15.02 ± 0.07	21.27 ± 0.03	40.5 ± 4.4
ER	0.39	0.50	1

**Table 7:** The LAC of nanoemulgel formulations and the control using porcine skin (mean ± SD, n = 3).

Parameters	MUP-NEG EO	MUP-NEG EU	Control
LAC (8 h)	3.74 ± 0.25	4.33 ± 0.42	1.45 ± 0.36
LAC (24 h)	0.91 ± 0.19	0.75 ± 0.39	0.87 ± 0.38

**Table 8:** The antibacterial activity of nanoemulgel against *S. aueus* and MRSA (n = 6, mean ± SD).

Formulations	Inhibition Zone Radius (mm)	
	*S. aureus* (NCIMB9518)	MRSA (NCTC13142)
Control	0	0
Bactroban cream	20.67 ± 1.03	21.67 ± 1.89
NEG EU	6.67 ± 0.52	6.17 ± 0.75
NEG EO	7.67 ± 0.52	8.33 ± 1.03
MUP-NEG EU	20.00 ± 2.37	22.17 ± 1.60
MUP-NEG EO	20.67 ± 0.52	20.83 ± 1.83

## Data Availability

Further details on the data can be found by contacting the corresponding author.

## Supplementary Materials

The following supporting information can be downloaded at: https://www.mdpi.com/article/10.3390/pharmaceutics15102387/s1, Figure S1: The flow profile of the control, hydrogel and nanoemulgel based on Carbopol (CBL) Xanthan gum (XG) and Hydroxypropyl methylcellulose (HPMC) (Mean ± SD, n = 3); Figure S2: The effect of polymer concentration on the rheological characteristics of hydrogels and nanoemulgels based on the polymer (A-B) Carbopol (C-D) Xanthan gum and (E) HPMC (Mean ± SD, n = 3); Figure S3: The effect of polymer concentration on the texture parameters of the gel and nanoemulgel formulations (A) Firmness; (B) Consistency; (C) Cohesiveness and (D) adhesiveness (Mean ± SD, n = 3); Table S1: The yield point value of hydrogel and nanoemulgel formulations.
