# Correction: Alhasso et al. Development of a Nanoemulgel for the Topical Application of Mupirocin. *Pharmaceutics* 2023, *15*, 2387

**DOI:** 10.3390/pharmaceutics16040448

**Published:** 2024-03-25

**Authors:** Bahjat Alhasso, Muhammad Usman Ghori, Simon P. Rout, Barbara R. Conway

**Affiliations:** 1 Department of Pharmacy, School of Applied Sciences, University of Huddersfield, Huddersfield HD1 3DH, UK; bahjat.alhasso@hud.ac.uk (B.A.); m.ghori@hud.ac.uk (M.U.G.); s.rout@hud.ac.uk (S.P.R.); 2 Department of Biological and Geographical Sciences, University of Huddersfield, Huddersfield HD1 3DH, UK; 3 Institute of Skin Integrity and Infection Prevention, University of Huddersfield, Huddersfield HD1 3DH, UK

## Addition of an Author

Simon P. Rout was not included as an author in the original publication [1]. In addition to affiliation 1, Simon P. Rout’s affiliations should include: ^2^ Department of Biological and Geographical Sciences, University of Huddersfield, Huddersfield HD1 3DH, UK. The corrected Author Contributions statement appears here. The authors state that the scientific conclusions are unaffected. This correction was approved by the Academic Editor. The original publication has also been updated.

**Author Contributions:** Conceptualization, B.R.C.; Methodology, B.A., M.U.G., S.P.R. and B.R.C.; Validation, B.A.; Formal analysis, B.A.; Investigation, B.A. and S.P.R.; Data curation, B.A.; Writing—original draft, B.A.; Writing—review and editing, B.R.C.; Supervision, M.U.G. and B.R.C.; Project administration, B.R.C. All authors have read and agreed to the published version of the manuscript.
