# Supplementary material for: Development of a Nanoemulgel for the Topical Application of Mupirocin
Source: Pharmaceutics. 2023 Sep 26;15(10):2387. doi: 10.3390/pharmaceutics15102387 (PMC10610056; doi:10.3390/pharmaceutics15102387)
Supplement: Supplementary file 1 [file pharmaceutics-15-02387-s001.zip › pharmaceutics-2603180-supplementary.pdf]

Supplementary materials

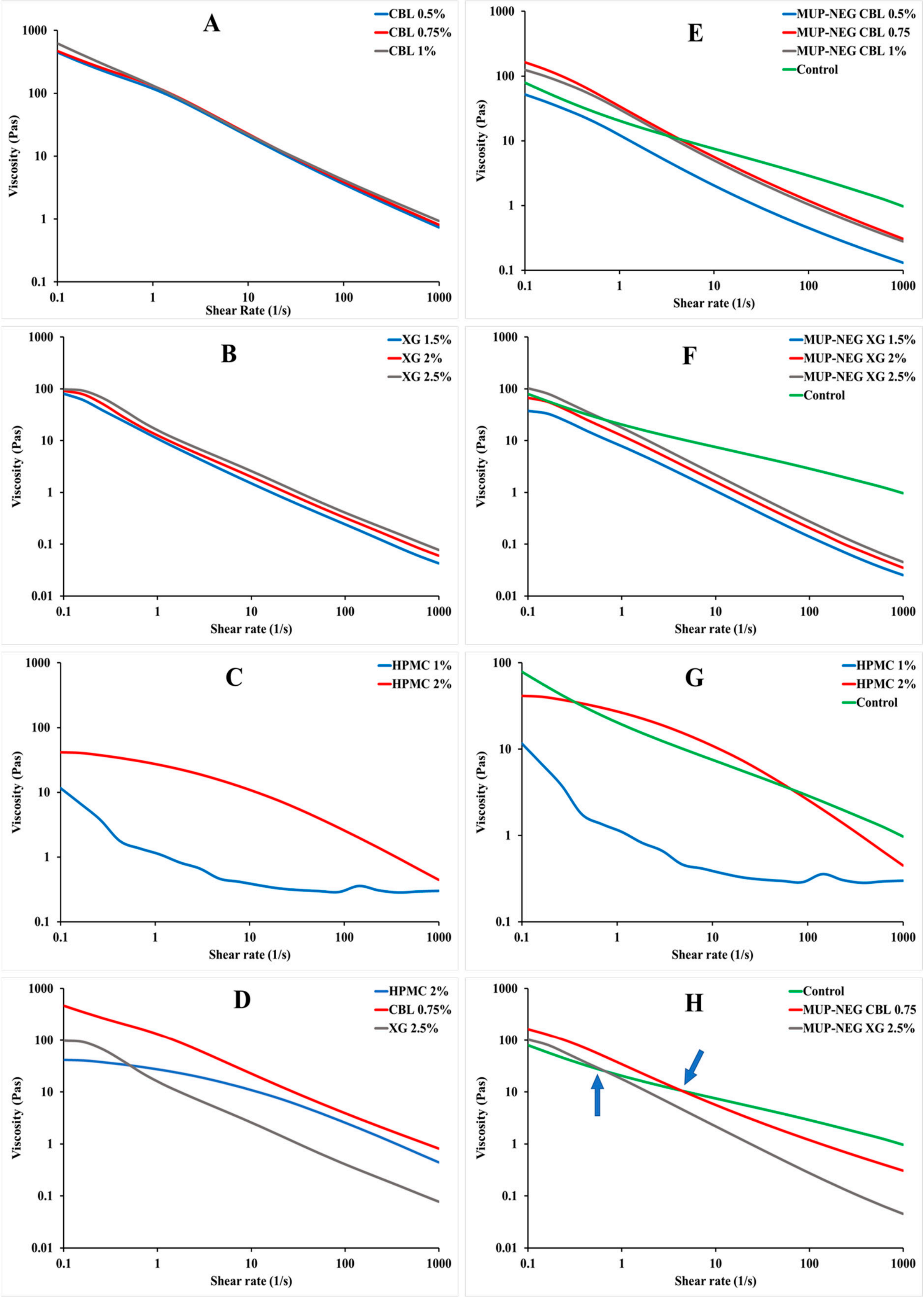

Figure S1 The flow profile of the control, hydrogel and nanoemulgel based on Carbopol (CBL) Xanthan gum (XG) and Hydroxypropyl methylcellulose (HPMC) (Mean ± SD, n=3).

Table S1 The yield point value of hydrogel and nanoemulgel formulations.

Hydrogel formulations	Yield point (Pa)	Nanoemulgel formulation	Yield point (Pa)
CBL 0.5%	428.59 ± 16.78	MUP-NEG CBL 0.5%	52.30 ± 0.26
CBL 0.75%	459.66 ± 27.10	MUP-NEG CBL 0.75%	140.33 ± 0.92
CBL 1%	496.12 ± 25.50	MUP-NEG CBL 1%	120.74 ± 1.52
XG 1.5%	25.83 ± 0.23	MUP-NEG XG 1.5%	14.45 ± 0.42
XG 2%	37.34 ± 1.02	MUP-NEG XG 2%	22.01 ± 0.47
XG 2.5%	48.56 ± 0.81	MUP-NEG XG 2.5%	28.87 ± 0.54
HPMC 1%	0.50 ± 0.21	Control	378.42 ± 6.78
HPMC 2%	313.05 ± 2.30		

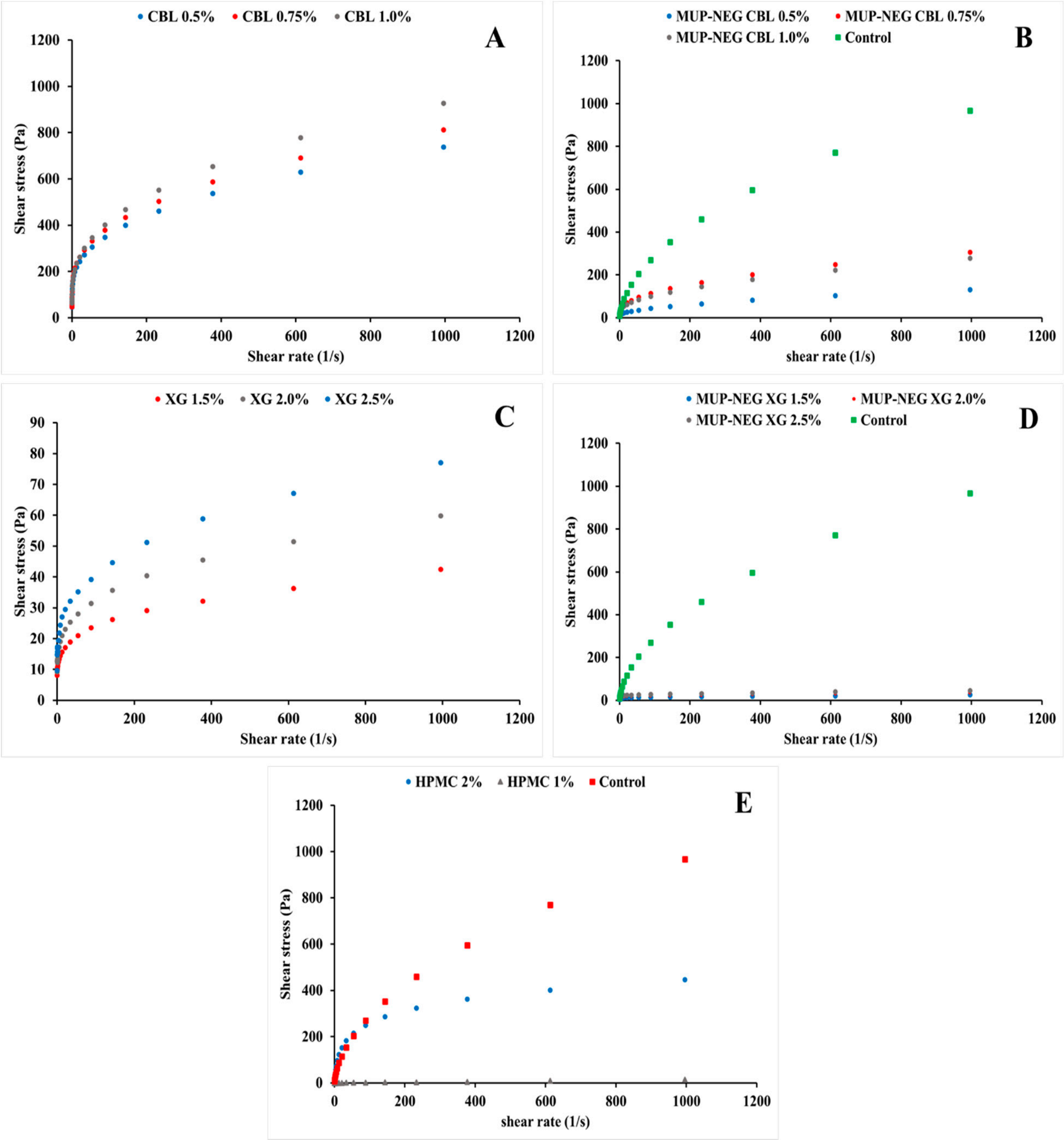

Figure S2 The effect of polymer concentration on the rheological characteristics of hydrogels and nanoemulgels based on the polymer (A-B) Carbopol (C-D) Xanthan gum and (E) HPMC (Mean ± SD, n=3).

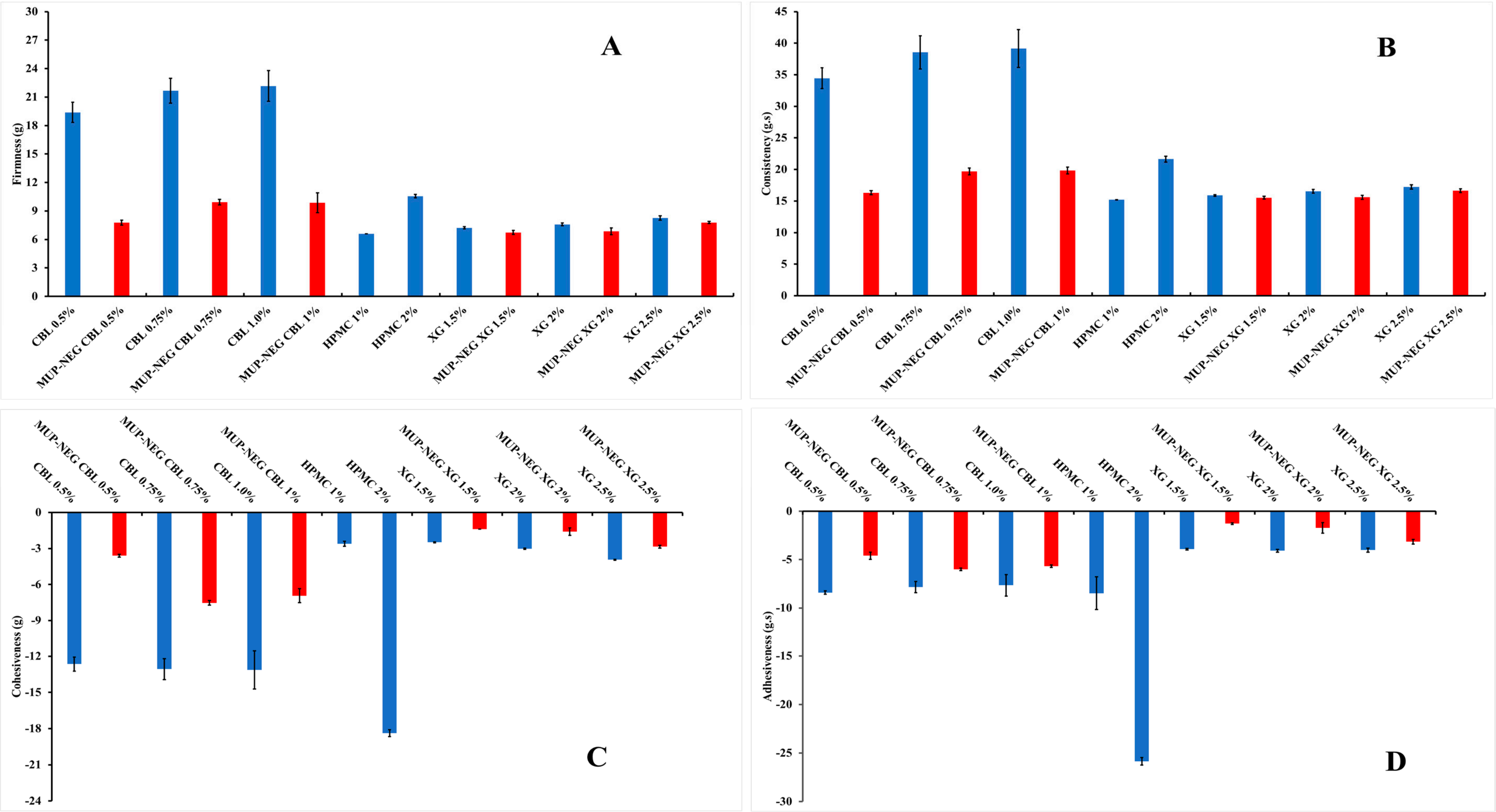

Figure S3 The effect of polymer concentration on the texture parameters of the gel and nanoemulgel formulations (A) Firmness; (B) Consistency; (C) Cohesiveness and (D) adhesiveness (Mean ± SD, n=3
